# Susceptibility Weighted Imaging as a Useful Imaging Adjunct in Hemichorea Hyperglycaemia

**DOI:** 10.1155/2013/456156

**Published:** 2013-05-23

**Authors:** Ferry Dharsono, Andrew Thompson, Jolandi van Heerden, Andrew Cheung

**Affiliations:** Neurological Intervention and Imaging Service of Western Australia, Sir Charles Gairdner Hospital, Hospital Avenue, Nedlands, WA 6009, Australia

## Abstract

Hyperglycaemia with hemichorea (HGHC) is an unusual clinical entity that can be associated with corpus striatum hyperintensity on T1-weighted (T1W) magnetic resonance imaging (MRI) sequences. We report the utility of the susceptibility weighted image (SWI) sequence and the filtered phase SWI sequence in the imaging assessment of HGHC.

## 1. Case Report

A 54-year-old man presented to the emergency department with a 5-day history of right-sided HemiChorea on a background of type II diabetes and hypertension. There was no personal or family history of movement disorders and no history of neuroleptic drug use. At presentation, serum blood glucose level was 36.2 mmol/L with an HbA1c of 16%. There was no ketonuria. The hemichorea resolved with optimal blood glucose control.

**Figure 1:**
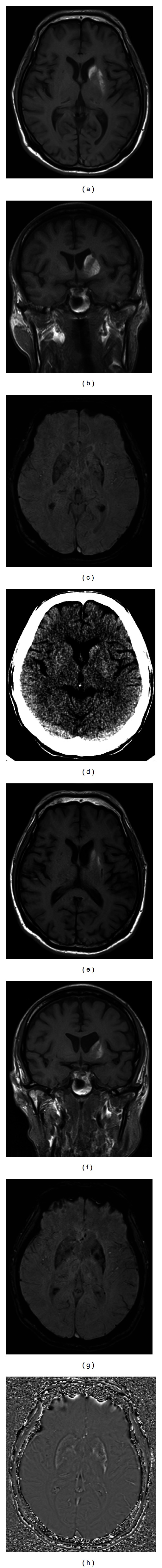
MRI changes in the left corpus striatum related to hemichorea hyperglycaemia. Top row: initial MRI head shows abnormal hyperintensity in the left caudate head and left putamen on axial and coronal T1W MRI (a) and (b). Asymmetric low signal was noted along the lateral margin of the left putamen on SWI (c). On NECT head (d) there is corresponding increased attenuation in the left corpus striatum. Bottom row: MRI head performed five months later shows volume loss of the entire left corpus striatum, cystic foci of malacic change in the posterolateral putamen along with partial resolution of the hyperintensity on axial and coronal T1W MRI (e) and (f). There was progression in extent and degree of hypointensity within the left corpus striatum on SWI (g) with corresponding hyperintensity on filtered phase SWI (h).

Initial nonenhanced CT (NECT) head demonstrated increased attenuation in the left corpus striatum, corresponding to hyperintensity on T1W MRI. There was no evidence of acute infarction on diffusion weighted imaging (DWI). The SWI sequence showed asymmetric hypointensity within the left lentiform nucleus, with sparing of the caudate nucleus (Figures 1(a)–1(d)). All MR studies were performed on a 1.5T superconducting MR imaging system (Aera; Siemens, Erlangen, Germany).

Surveillance MRI at five months revealed volume loss of the entire left corpus striatum with partial resolution of the hyperintensity on T1W MRI, particularly at the caudate head. Small cystic foci of malacic change were demonstrated within the left posterolateral putamen. The SWI sequence revealed interval progression in the extent and degree of hypointensity in the left lentiform nucleus and caudate head. SWI hypointensity corresponded to areas of hyperintensity on the filtered phase SWI sequence (Figures 1(e)–1(h)). These findings are consistent with deposition of paramagnetic material in a left-handed coil MR system, such as the Aera (Siemens; Erlangen, Germany).

## 2. Discussion

Hemichorea is defined as continuous, nonrhythmic, involuntary movements affecting one side of the body. A focal vascular insult to the contralateral basal ganglia is considered to be the most common cause. Nonketotic hyperglycemia is a less usual cause for this presentation [1–3]. Affected patients are typically poorly controlled diabetics who experience acute-onset Hemichorea, hemiballismus, and occasionally, altered mental status [4], with imaging often demonstrating characteristic CT high attenuation of one or both corpus striati with associated abnormal hyperintensity on T1W MRI [5, 6].

The pathophysiological basis for striatal signal alteration in HGHC is unclear; however various hypotheses exist [4]. Some authors suggest microhaemorrhage as a cause [7], while other studies failed to demonstrate microhaemorrhage histopathologically, suggesting calcium deposition as the favoured aetiology [3]. Others consider cerebral ischemia as the most appropriate explanation of the T1 signal alteration [8, 9]. 

Histopathologically, Shan and coauthors reported the presence of abundant gemistocytes (reactive swollen astrocytes) without evidence of hemorrhage [3]. Ohara et al. described autopsy-proven lacunar infarcts associated with reactive astrocytosis within the affected putamen [10] and Neal et al. showed mineral deposition in hypertrophied astrocytes located within ischaemic brain [11]. A case report by Cherian et al. suggests paramagnetic mineral deposition in the affected putamen caused by swollen gemistocytes that express metallothionein and zinc secondary to ischaemic insult [1]. The hypothesis of transient ischaemia was supported by Mittal who described the presence of ipsilateral prominent cortical veins on SWI sequences [12].

Our case report explores the utility of the final SWI and filtered phase SWI sequences in identifying the nature of the striatal hyperintensity. 

SWI is a relatively recent MR technique that exploits susceptibility differences between tissues by using both magnitude and phase images from a high resolution 3D fully velocity compensated radio-frequency (RF) spoiled gradient echo sequence. The phase image, which detects differences between tissues, is corrected by applying a high pass filter to remove unwanted artefacts. A phase mask is created from the corrected phase image and the final SWI sequence is created by multiplying the magnitude images with the phase mask [13].

The final SWI sequence utilizes the susceptibility differences between adjacent tissues to produce tissue contrast and is extremely sensitive in the detection of diamagnetic and paramagnetic substances such as blood products, calcium, iron, free radicals from macrophage phagocytosis, and small vessels [13–16].

Although the final SWI sequence demonstrates low signal for both para- and diamagnetic substances, filtered phase sequences more accurately depict subtle differences in substance susceptibility [14, 15]. On phase sequences paramagnetic substances, including free radicals, blood products, and iron, exhibit high signal, in contrast to diamagnetic substances such as calcium which return low signal [14–16]. 

In this case, MRI was performed on admission with subsequent MRI five months later. Initial SWI revealed hypointensity in the left lentiform nucleus only, with disproportionately more extensive hyperintensity on T1W MRI. Follow-up imaging five months later showed volume loss associated with improving hyperintensity on T1W MRI and more extensive and increased SWI hypointensity within the affected corpus striatum. The SWI sequence changes corresponded to areas of high signal on phase filtered SWI sequences. This suggests interval deposition of paramagnetic material, which could represent either mineralization or blood [13–15]. 

In our opinion, hemorrhage is unlikely based on the disproportionate extensive hyperintensity on the initial T1W sequence with comparatively little SWI hypointensity [1]. Interval progression of SWI hypointense signal within the left caudate nucleus also suggests an ongoing process of deposition rather than a single initial haemorrhagic event. 

We postulate that the deposited paramagnetic material is most likely iron, accounting for the NECT head high attenuation. MRI imaging features further support this hypothesis, explaining the T1W MRI hyperintensity and SWI hypointensity and corresponding filtered phase SWI hyperintensity [17]. The progressive left corpus striatum malacic change demonstrated on follow-up imaging can be explained by iron-deposition-related neurotoxicity. The partial resolution of T1W hyperintensity at subsequent MRI with concomitant increased SWI hypointensity may reflect changes in the paramagnetic properties of iron during degradation, with resultant variable signal characteristics on T1W sequences. 

## 3. Summary

Hemichorea hyperglycaemia is a rare syndrome often associated with hyperintensity on T1W imaging affecting the corpus striatum. We correlated this signal abnormality with the phase filtered SWI sequence to better define the nature of the corpus striatum signal change. We propose that deposition of paramagnetic material, in particular iron, is most likely responsible for the MRI signal abnormality associated with this condition.
